# eHealth solutions for trans* persons: a systematic literature review of research from 2000 till 2021: Susanne Gahbauer

**DOI:** 10.1093/eurpub/ckac131.508

**Published:** 2022-10-25

**Authors:** S Gahbauer, D Haluza

**Affiliations:** 1 Department of Social and Preventive Medicine, Medical University of Vienna, Vienna, Austria; 2 Department of Environmental Health, Medical University of Vienna, Vienna, Austria

## Abstract

Though familiarity with trans* personś needs has increased in the new millennium, still, addressing their specific health issues is challenging. In and outside of complex interdisciplinary settings with trans* persons and health care workers, ICT solutions are used to remind trans* persons of their hormone injection or to conduct voice training. Hence, we reviewed the pertinent literature to shed light on what kind of eHealth solutions are investigated, what diagnosis and treatment are eHealth solutions related to, and how are trans* persons enacted within these eHealth solutions. We conducted a systematic literature review analyzing peer reviewed articles presenting all kind of studies except literature reviews. Using the electronic databases PubMed and Scopus, we analyzed data from 1 January 2000 to 31 December 2021. For screening purposes we used the PRISMA checklist and, data extraction followed the PICO (Population, Intervention, Control, Outcome, and Improvement) model. We also analyzed studies covering aspects of the impact on the COVID-19 pandemic. Overall, we identified 322 records, 187 from Scopus and 135 from PubMed. Removing 117 duplicated and 47 records for other reasons, 158 reports were assessed for eligibility. Our results show that trans* persons were often presented as subsamples in larger samples of non-binary populations. Also, eHealth solutions related to very different technological solutions and to a wide range of treatment models, with the vast majority relating to sexual health. Hence, we could also see that trans* women were the more interesting group in the research included in this review. We found 23 studies relating to COVID-19. eHealth solutions have great potential to contribute to a better healthcare for trans* persons but the needs of different groups during healthcare have to be taken into account in further research. During the COVID-19 pandemic, the need for research on eHealth solutions for trans* persons came into focus.

**Key messages:**

• Our analyzes showed that research on eHealth solutions for trans* persons takes place in complex interdisciplinary settings which need to be taken into account in further research.

• The COVID-19 pandemic crisis lead to an uptake of eHealth interventions in mental, endocrinological, and sexual health care, especially in younger groups and in the frame of routine clinical care.

